# A key to the East Palaearctic and Oriental species of the genus *Rhysipolis* Foerster, and the first host records of *Rhysipolis longicaudatus* Belokobylskij (Hymenoptera: Braconidae: Rhysipolinae)

**DOI:** 10.3897/BDJ.4.e7944

**Published:** 2016-05-04

**Authors:** Ying Zhang, Zi-Cheng Xiong, Kees van Achterberg, Tao Li

**Affiliations:** ‡ General Station of Forest Pest Management, State Forestry Administration, Shenyang 110034, China; § Forest Pest Control and Quarantine Station of Etuoke, Inner Mongolia Autonomous Region, China; | Department of Life Sciences, Northwest University, 229 North Taibai Road, Xi’an, China; ¶ Department of Terrestrial Zoology, Naturalis Biodiversity Center, Leiden, Netherlands

**Keywords:** Rhysipolinae, Psychidae, Pyralidae, *Rhysipolis longicaudatus*, new hosts, parasitoid

## Abstract

**Background:**

A key to the East Palaearctic and northern Oriental species of *Rhysipolis* Foerster, 1862 (Hymenoptera: Braconidae: Rhysipolinae) is presented. *Rhysipolis longicaudatus* Belokobylskij, 1994 (**stat. nov.**) is redescribed, the first host records are given and it is reported new for China.

**New information:**

*Rhysipolis longicaudatus* was reared from *Taleporia* sp. (Lepidoptera: Psychidae) in Inner Mongolia Autonomous Region and from *Bazaria turensis* Ragonot (Lepidoptera: Pyralidae) in Qinghai Province.

## Introduction

The genus *Rhysipolis* Foerster, 1862 (Hymenoptera: Braconidae: Rhysipolinae) includes 22 described species (Yu et al. 2012), of which nine species from the Palaearctic region (Belokobylskij 1985, Belokobylskij 1986, Haliday 1836, Papp 1987, Tobias 1976, Yu et al. 2012), eight species from the Nearctic region (one of them up to the Neotropical region) (Spencer and Whitfield 1999), three species from the Neotropical region (Scatolini et al. 2002, Spencer and Whitfield 1999), four species from the Oriental region (Belokobylskij 1988, Belokobylskij and Vu 1988), and one species from the Afrotropical region (Cape Verde Islands, Papp 1996). We present the first key to the East Palaearctic and Oriental species of the genus *Rhysipolis* Foerster to allow the identification of the reared Chinese specimens.The key can be used as a basis for revising the Chinese *Rhysipolis* species.

The recorded hosts of *Rhysipolis* species mainly belong to the lepidopteran families Gelechiidae and Gracillariidae, but also Choreutidae, Cosmopterigidae, Elachistidae, Hesperiidae, Lyonetiidae, Momphidae, Pyralidae, Tineidae, Tischeriidae and Tortricidae have been reported (Belokobylskij 1988, Gates et al. 2002, Marczak and Buszko 1994, Shaw 1983, Shaw and Askew 1976, Spencer and Whitfield 1999). In this paper, we report *Taleporia* sp. (Lepidoptera: Psychidae) and *Bazaria turensis* Ragonot (Lepidoptera: Pyralidae) as hosts of *Rhysipolis longicaudatus* Belokobylskij, 1994 (Belokobylskij 1994) (**stat. nov.**). The isolated records of non-lepidopterous hosts (Curculionidae, Bostrichidae, Tephritidae, Otitidae and Anthomyiidae) need confirmation.

## Materials and methods

Mature larvae of a bagworm moth, *Taleporia* sp. (Lepidoptera: Psychidae) were collected in Etuoke (Inner Mongolia Autonomous Region), and larvae of *Bazaria turensis* Ragonot (Lepidoptera: Pyralidae) in Dulan County, Qinghai Province, NW China. The larvae were brought to the laboratory and maintained in a large nylon cage at room temperature. All larvae were checked daily for pupation and parasitoid emergence. Emerged parasitoid larvae and pupae were kept individually in glass tubes (100 mm long and 15 mm in diameter) with a piece of filter paper dipped in distilled water in order to prevent desiccation and plugged with absorbent cotton. The hosts were identified by Prof. Hou-Hun Li (Nankai University, Tianjin).

For the morphological terminology used in this paper see van Achterberg (1993) and Harris (1979). The descriptions, measurements and figures were made with a Leica M205A microscope (Leica Microsystems (Switzerland) Limited). Focused photographs were combined using Leica DFC550 with Leica Application Suite (Version 4.5.0).

The specimens and hosts are deposited in the Insect Museum, General Station of Forest Pest Management (GSFPM), State Forestry Administration, Shenyang, P. R. China, and Naturalis Biodiversity Center (RMNH), Leiden, the Netherlands.

## Taxon treatments

### Rhysipolis longicaudatus

Belokobylskij, 1994

#### Materials

- **Type status:** Other material. **Occurrence:** occurrenceRemarks: Reared from the bagworm moth genus Taleporia (Lepidoptera: Psychidae) on Caragana korshinskii Kom. (Leguminosae); recordedBy: Mao-Ling Sheng; individualCount: 1; sex: female; **Location:** country: NW. China; locality: Inner Mongolia Autonomous Region, Etuoke; **Event:** eventDate: 10/05/2014
- **Type status:** Other material. **Occurrence:** occurrenceRemarks: Reared from the bagworm moth genus Taleporia (Lepidoptera: Psychidae) on Caragana korshinskii Kom. (Leguminosae); recordedBy: Mao-Ling Sheng; individualCount: 2; sex: male; **Location:** country: NW. China; locality: Inner Mongolia Autonomous Region, Etuoke; **Event:** eventDate: 10/02/2014
- **Type status:** Other material. **Occurrence:** occurrenceRemarks: Reared from the bagworm moth genus Taleporia (Lepidoptera: Psychidae) on Caragana korshinskii Kom. (Leguminosae); recordedBy: Mao-Ling Sheng; individualCount: 2; sex: 1 male, 1 female; **Location:** country: NW. China; locality: Inner Mongolia Autonomous Region, Etuoke; **Event:** eventDate: 10/03/2014
- **Type status:** Other material. **Occurrence:** occurrenceRemarks: Reared from the bagworm moth genus Taleporia (Lepidoptera: Psychidae) on Caragana korshinskii Kom. (Leguminosae); recordedBy: Mao-Ling Sheng; individualCount: 1; sex: male; **Location:** country: NW. China; locality: Inner Mongolia Autonomous Region, Etuoke; **Event:** eventDate: 10/04/2014
- **Type status:** Other material. **Occurrence:** occurrenceRemarks: Reared from the bagworm moth genus Taleporia (Lepidoptera: Psychidae) on Caragana korshinskii Kom. (Leguminosae); recordedBy: Mao-Ling Sheng; individualCount: 4; sex: 1 female, 3 male; **Location:** country: NW. China; locality: Inner Mongolia Autonomous Region, Etuoke; **Event:** eventDate: 10/05/2014
- **Type status:** Other material. **Occurrence:** occurrenceRemarks: Reared from the bagworm moth genus Taleporia (Lepidoptera: Psychidae) on Caragana korshinskii Kom. (Leguminosae); recordedBy: Mao-Ling Sheng; individualCount: 1; sex: female; **Location:** country: NW. China; locality: Inner Mongolia Autonomous Region, Etuoke; **Event:** eventDate: 10/08/2014
- **Type status:** Other material. **Occurrence:** occurrenceRemarks: Reared from the bagworm moth genus Taleporia (Lepidoptera: Psychidae) on Caragana korshinskii Kom. (Leguminosae); recordedBy: Mao-Ling Sheng; individualCount: 2; sex: male; **Location:** country: NW. China; locality: Inner Mongolia Autonomous Region, Etuoke; **Event:** eventDate: 10/07/2014
- **Type status:** Other material. **Occurrence:** occurrenceRemarks: Reared from the bagworm moth genus Taleporia (Lepidoptera: Psychidae) on Caragana korshinskii Kom. (Leguminosae); recordedBy: Mao-Ling Sheng; individualCount: 2; sex: 1 male, 1 female; **Location:** country: NW. China; locality: Inner Mongolia Autonomous Region, Etuoke; **Event:** eventDate: 10/09/2014
- **Type status:** Other material. **Occurrence:** occurrenceRemarks: Reared from the bagworm moth genus Taleporia (Lepidoptera: Psychidae) on Caragana korshinskii Kom. (Leguminosae); recordedBy: Mao-Ling Sheng; individualCount: 1; sex: female; **Location:** country: NW. China; locality: Inner Mongolia Autonomous Region, Etuoke; **Event:** eventDate: 10/10/2014
- **Type status:** Other material. **Occurrence:** occurrenceRemarks: Reared from the bagworm moth genus Taleporia (Lepidoptera: Psychidae) on Caragana korshinskii Kom. (Leguminosae); recordedBy: Mao-Ling Sheng; individualCount: 4; sex: 3 female, 1 male; **Location:** country: NW. China; locality: Inner Mongolia Autonomous Region, Etuoke; **Event:** eventDate: 10/11/2014
- **Type status:** Other material. **Occurrence:** occurrenceRemarks: Reared from the bagworm moth genus Taleporia (Lepidoptera: Psychidae) on Caragana korshinskii Kom. (Leguminosae); recordedBy: Mao-Ling Sheng; individualCount: 3; sex: female; **Location:** country: NW. China; locality: Inner Mongolia Autonomous Region, Etuoke; **Event:** eventDate: 10/13/2014
- **Type status:** Other material. **Occurrence:** occurrenceRemarks: Reared from the bagworm moth genus Taleporia (Lepidoptera: Psychidae) on Caragana korshinskii Kom. (Leguminosae); recordedBy: Mao-Ling Sheng; individualCount: 1; sex: female; **Location:** country: NW. China; locality: Inner Mongolia Autonomous Region, Etuoke; **Event:** eventDate: 10/15/2014
- **Type status:** Other material. **Occurrence:** occurrenceRemarks: Reared from Bazaria turensis Ragonot (Lepidoptera: Pyralidae) on Nitraria sp. (Zygophyllaceae); recordedBy: Mao-Ling Sheng; individualCount: 3; sex: female; **Location:** country: NW. China; locality: Qinghai Province, Dulan County; **Event:** eventDate: 09/30/2014
- **Type status:** Other material. **Occurrence:** occurrenceRemarks: Reared from Bazaria turensis Ragonot (Lepidoptera: Pyralidae) on Nitraria sp. (Zygophyllaceae); recordedBy: Mao-Ling Sheng; individualCount: 1; sex: female; **Location:** country: NW. China; locality: Qinghai Province, Dulan County; **Event:** eventDate: 10/09/2014
- **Type status:** Other material. **Occurrence:** occurrenceRemarks: Reared from Bazaria turensis Ragonot (Lepidoptera: Pyralidae) on Nitraria sp. (Zygophyllaceae); recordedBy: Mao-Ling Sheng; individualCount: 1; sex: male; **Location:** country: NW. China; locality: Qinghai Province, Dulan County; **Event:** eventDate: 10/07/2014

#### Description

**Figure 1.**
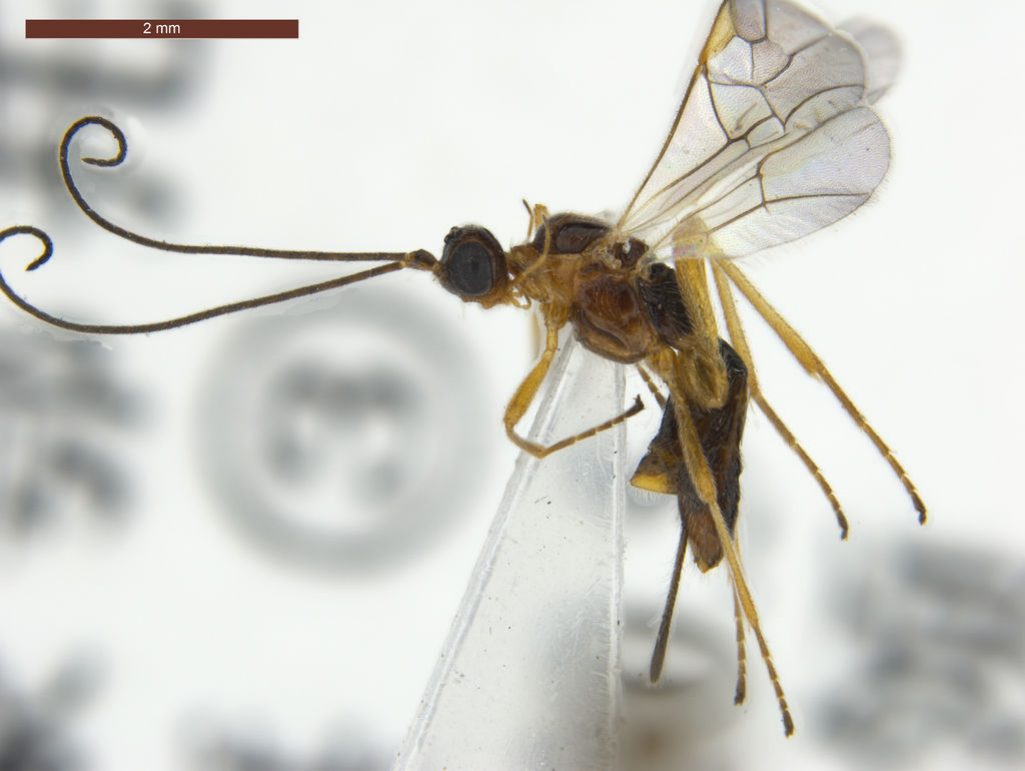
*Rhysipolis longicaudatus* Belokobylskij, female, China, Etuoke. Habitus, lateral view.

**Figure 3.**
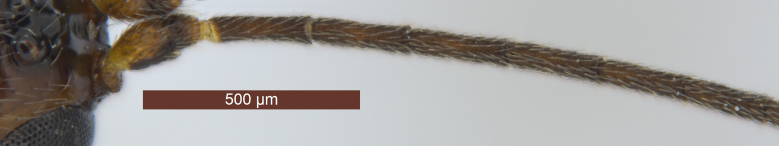
*Rhysipolis longicaudatus* Belokobylskij, female, China, Etuoke. Basal antennal segments.

**Female.** Length of body 2.7–4.0 mm, and of fore wing 2.9–4.0 mm (Figs 1, 2, 3, 4, 5, 6).

**Head** (Fig. 2). Head width 1.6 × its median length in dorsal view, 1.1 × width of mesoscutum, smooth with uniformly white setae. Temple behind eye (dorsal view) roundly narrowed. Frons (Fig. 2b) flat and smooth, medially weakly concave, sparsely white setae along the eyes. Ocelli medium-sized and near stemmaticum distinctly concave. POL 1.1 × Od, 0.5 × OOL. Eye glabrous, slightly concave near antennal sockets, 1.3 × as high as wide, 3.1 × temple in dorsal view. Diameter of antennal sockets 0.9 × distance between antennal sockets, 1.7 × distance between socket and eye. Face (Fig. 2a) smooth with long white setae and oblique rugae below sockets; width of face 0.8 × height of eye and 1.2 × height of face and clypeus combined. Length of malar space 0.2 × height of eye, 0.8 × basal width of mandible. Malar suture absent. Clypeus suture distinct. Hypoclypeal depression round, 1.9 × as wide as distance from depression to eye and 0.5 × as wide as face. Occipital carina complete dorsally, joining hypostomal carina at base of mandible. Antenna (Fig. 3) with 28-34 segments, setiform and slender, 1.1–1.2 × longer than body. Scape 1.5 × longer than its maximum width. Third segment 3.5 × longer than its apical width, 0.9–1.0 × fourth segment. Penultimate segment 1.9 × longer than its maximum width, 0.8 × longer than apical segment.

**Mesosoma** (Figs 4a, 5a). Length of mesosoma 1.7 × longer than high. Pronotum convex laterally with irregular wrinkles. Median lobe of mesoscutum (Fig. 4a) distinctly convex, smooth with uniformly long white setae; median portion in posterior with fine longitudinal carinae. Most portion of lateral lobe glabrous. Notauli with fine wrinkles, deep in anterior half and shallow in posterior half. Scutellar sulcus wide, with a high median carina, smooth, 0.3–0.4 × as long as scutellum. Scutellum flat, smooth with uniformly white setae. Metanotum conspicuously concave, with short wrinkles. Upper portion of mesopleuron (Fig. 5a) with obvious wrinkles and white setae, most of median portion glabrous, white setae in posterior area; speculum concave; prepectal carina complete, joining with anterior of mesopleuron; precoxal sulcus distinct with fine crenulate. Propodeum (Fig. 4a) roundly convex, median smooth except for anterior and posterior rugose areas; median carina strong, 0.6 × as long as propodeum, lateral areas of median carina obviously concave with transverse wrinkles.

**Wings** (Fig. 6). Fore wing (Fig. 6a): length about 3 × as long as its maximum width. Pterostigma 5.3–5.4 × as long as its maximum width. Vein M+CU1 straight; vein r-m present; vein 1-SR+M almost straight; vein r arising behind middle of pterostigma; 2-SR 2.3 × as long as r, 0.7 × as long as 3-SR, 0.5 × as long as SR1, 1.9–2.0 × as long as r-m; 1-CU1 0.3–0.4 × as long as 2-CU1. Hind wing (Fig. 6b): vein M+CU1 almost straight; 1-M 1.1–1.3 × as long as M+CU1; vein SR present; vein 3-M obvious and straight.

**Legs** (Figs 1, 5b). Hind coxa 1.9–2.0 × as long as wide, smooth with long white setae; hind femur 5.3–5.6 × as long as wide; hind tarsus 0.9 × as long as hind tibia; basitarsus 0.6–0.7 × as long as second-fifth segments combined; second segment of hind tarsus 0.5–0.6 × as long as basitarsus, 1.7–1.8 × as long as fifth segment (without arolium).

**Metasoma** (Fig. 4b). Length 0.8–0.9 × as long as head and mesosoma combined. Dorsope large, triangular. Apical half of first tergite uniformly convex, with finely striate and transverse sculpture between striae; apical margin with obviously oblique wrinkles; dorsal carinae strong, distinctly converging, length 0.5–0.6 × as long as first tergite; apical width 2.0–2.3 × as long as its basal width; length of first tergite 0.9–1.0 × as long as its apical width. Remaining tergites smooth, with uniformly white setae. Hypopygium large, triangular. Ovipositor sheath 0.6–0.7 × as long as metasoma and 0.3 × as long as fore wing (Fig. 1).

**Colour.** Dark brown to blackish brown (Fig. 1). Mandible (apically dark brown), palpi, pronotum, legs (but telotarsus and claws dark brown), tegulae and pterostigma brownish yellow; middle lobe of mesoscutum, scutellum and mesopleuron, dark brown with reddish brown pattern; wing membrane subhyaline, veins brown. Alternatively, head (but stemmaticum dark brown), mesosoma (propodeum blackish brown), palpi, legs (telotarsus and claws brown or dark brown), tegula and pterostigma yellowish brown; antenna dark brown; metasoma (but apical tergite yellowish brown) blackish brown. Second-fifth tergites laterally and sixth tergite may be yellowish brown.

**Male.** Length of body 2.5–3.1 mm, and of fore wing 2.2–3.2 mm. Antennal segments 28–33, length 2.6–4.0 mm. Length of mesosoma 2.0–2.6 × its height. Length of first tergite 1.0–1.2 × as long as its apical width and apical width 1.9–2.0 × as long as its basal width. Head, metasoma and antenna, dark brown; palpi, mesosoma (propodeum brown), legs (telotarsus and claw brown), tegula, pterostigma and veins, yellowish brown. Alternatively, face (median with little reddish brown), mandible (apical black brown), palpi, pronotum, legs (tarsus and claw, brown), tegulae, pterostigma, vein, yellowish brown; antenna (ventral of scope yellowish brown), dark brown; vertex, frons (lateral margin dark reddish brown), temple, scutellar sulcus, precoxal sulcus, dark reddish brown to dark brown; metanotum, propodeum and first tergite, blackish brown or first tergite dark yellowish brown and remaining tergites dark brown.

#### Diagnosis

Setose part of ovipositor sheath about 0.7 × as long as hind tibia (its total length about 1.3 × tibia); clypeus and mesoscutum medially yellowish, with face and mesoscutum laterally dark brown; propodeum with closed, parallel-sided and laterally lamelliform areola, sculptured medio-posteriorly, without semicircular smooth and shiny convex area; notauli present posteriorly (Fig. 4a); mesoscutum finely sculptured medio-posteriorly; vein M+CU of hind wing 0.8–1.0 × as long as vein 1-M; mesoscutum steep anteriorly. This species was described by Belokobylskij (1994) as a subspecies of *R. meditator* (Haliday). It is treated here as a valid species because the differences in the ovipositor length, the shorter temples, the colour of the clypeus and the mesoscutum and the shape of the propodeal areola indicate that it is a separate species. Colour and shape of the propodeal areola are similar in *R. setmus* Papp, but the latter species has the head and the mesoscutum entirely brownish yellow, the first tergite largely smooth and a shorter ovipositor sheath.

#### Distribution

NW China: Inner Mongolia Autonomous Region; Qinghai (new record for China); Far East Russia.

#### Biology

Reared from the larvae of *Taleporia* sp. (Lepidoptera: Psychidae) on *Caragana korshinskii* Kom. (Leguminosae) and from *Bazaria turensis* Ragonot (Lepidoptera: Pyralidae) on *Nitraria* sp. (Zygophyllaceae). First host records.

## Identification Keys

### Key to East Palaearctic and Oriental species of the genus *Rhysipolis* Foerster

**Table:** 

1	Vein 3-SR of fore wing 1.6–1.8 × longer than vein r-m and 1.2–1.3 × as long as vein 2-SR; hypoclypeal depression wide elliptical and about 0.8 × minimum width of face; hind femur 3.3–3.6 × as long as wide	***R. mongolicus*** Belokobylskij, 1985
–	Vein 3-SR of fore wing 2.3–3.0 × longer than vein r-m and 1.4–1.7× as long as vein 2-SR; hypoclypeal depression semicircular and 0.4–0.7 × minimum width of face; hind femur 4.3–5.5 × as long as wide	2
2	Clypeus close to eyes, distance between anterior tentorial pit and eye about equal to width of tentorial pit; head distinctly transverse in dorsal view; hypoclypeal depression 0.7 × as wide as face; malar space 0.2–0.3 × basal width of mandible; eye in dorsal view about 4.0 × as long as temple; OOL 0.5–0.8 × diameter of posterior ocellus	***R. oculator*** Belokobylskij, 1986
–	Clypeus distinctly removed from eyes, distance between anterior tentorial pit and eye at least 3.0 × width of tentorial pit; head trapezoid in dorsal view; hypoclypeal depression 0.4–0.6 × as wide as face; malar space 0.4–0.7 × basal width of mandible; eye in dorsal view 1.6–3.8 × as long as temple; if more than 3.0 × then OOL about twice diameter of posterior ocellus	3
3	Pronotum with pair of slightly converging carinae submedially; dorsal part of propodeum of male densely granulate; [ovipositor sheath slightly longer than first tergite; notauli crenulate anteriorly]	***R. bicarinator*** Belokobylskij, 1986
–	Pronotum without pair of submedial carinae or nearly so, but distinctly converging carinae may be present sublaterally; propodeum at least partly smooth	4
4	Propodeum with distinct semicircular smooth and strongly shiny convex area medio-posteriorly; pronotum comparatively large and wide anteriorly; notauli absent posteriorly, only anteriorly narrowly impressed; mesoscutum smooth medio-posteriorly or sculptured; vein M+CU of hind wing 0.6–0.8 × vein 1-M; *R. decorator*-group	5
–	Propodeum sculptured medio-posteriorly, without semicircular smooth and shiny convex area (Fig. 4a) or shiny area very narrowly developed; pronotum medium-sized and narrower anteriorly (Fig. 4a); notauli usually complete, but narrowly impressed posteriorly (Fig. 4a); mesoscutum usually finely sculptured medio-posteriorly; vein M+CU of hind wing 0.8–1.0 × vein 1-M; *R. meditator*-group [= *Rhysipolis* s.s.]	8
5	Dorsal margin of clypeus near lower level of eyes; eye in dorsal view about twice as long as temple **and** no long setae near occipital carina; [notauli crenulate anteriorly; pronope obsolescent]	***R. enukidzei*** Tobias, 1976 Syn.: *R. alacer* Papp, 1987
–	Dorsal margin of clypeus distinctly above lower level of eyes; eye in dorsal view 1.8–3.8 × as long as temple, **if** 1.8–2.4 × then with long setae near occipital carina; [pronotum with distinct anterior lamelliform rim]	6
6	Occipital carina with fringe of long curved setae (Fig. 2b); length of eye in dorsal view 1.8–2.4 × temple; propleuron often black anteriorly, contrasting with yellowish pronotum; [width of face about 1.7 × width of hypoclypeal depression; mesoscutum smooth medio-posteriorly]	***R. hariolator*** (Haliday, 1836) Syn.: *R. barbatus* (Wesmael, 1838)
–	Occipital carina without fringe of long setae; length of eye in dorsal view 3.5–3.8 × temple; propleuron yellowish anteriorly, hardly or not contrasting with most of pronotum (Fig. 5a)​; [length of ovipositor sheath 0.7–0.8 × as long as hind tibia]	7
7	Notauli smooth anteriorly or nearly so; face brownish-yellow and smooth laterally; medio-dorsally pronotum dark brown or infuscate and weakly contrasting with dark brown or black mesoscutum; [mesoscutum smooth medio-posteriorly; eyes oval (Fig. 2a); hind tibia and hind tarsus largely dark brown]	***R. decorator*** (Haliday, 1836) Syn.: *R. ruficeps* (Wesmael, 1838); *R. ruficornis* (Szépligeti, 1896); *R. caudatus* (Thomson, 1892)
–	Notauli crenulate anteriorly; face black or mainly dark brown and usually laterally micro-sculptured; medio-dorsally pronotum striking yellow and strongly contrasting with black mesoscutum; [temples directly narrowed in dorsal view; shallow pronope present]	***R. temporalis*** Belokobylskij, 1986 Syn.: *R. geranus* Papp, 1987
8	Setose part of ovipositor sheath 0.2–0.3 × as long as hind tibia (total length 0.4–0.5 × tibia) and 1.0–1.3 × as long as first tergite; [clypeus, face and mesoscutum entirely brownish yellow or dark brown]	9
–	Setose part of ovipositor sheath 0.6–0.7 × as long as hind tibia (total length up to 1.3 × tibia) and 2.0–3.0 × as long as first tergite	12
9	Stemmaticum minute, width of frons about 3.5 × width of stemmaticum; middle lobe of mesoscutum glabrous; [head strongly narrowed behind eyes and subglobular]	***R. taiwanicus*** Belokobylskij, 1988
–	Stemmaticum medium-sized, width of frons about 2.5 × width of stemmaticum; middle lobe of mesoscutum setose; head roundly narrowed behind eyes	10
10	Vein r of fore wing strongly oblique, distal angle with pterostigma about 60º; marginal cell of fore wing about 4.0 × as long as its maximum width; Oriental	***R. parnarae*** Belokobylskij & Vu, 1988
–	Vein r of fore wing moderately oblique, distal angle with pterostigma nearly 90º (Fig. 6a); marginal cell of fore wing 3.0–3.5 × as long as its maximum width; Palaearctic	11
11	Clypeus, face, pronotum anteriorly and mesoscutum brownish yellow; lateral carinae of propodeal areola parallel-sided and areola closed anteriorly; notauli moderately wide and distinctly crenulate; first tergite largely smooth; pterostigma yellow	***R. setmus*** Papp, 1987
–	Clypeus, face, pronotum anteriorly and mesoscutum black or blackish; lateral carinae of propodeal areola diverging anteriorly and areola open anteriorly; notauli narrow and finely crenulate anteriorly; first tergite largely sculptured; pterostigma largely rather dark brown; [middle lobe of mesoscutum with shallow median groove, glabrous and strongly shiny; malar space 0.2 × as long as height of eye]	***R. meditator*** (Haliday, 1836) Syn.: *R. decorator* auctt. p.p.; *R. intermedius* (Wesmael, 1838); R. meditator f. brevicaudatus Belokobylskij, 1994
12	Mesoscutum finely granulate; medially ventral rim of clypeus far below lower level of eyes	***R. townesi*** Belokobylskij, 1988
–	Mesoscutum (except some rugosity medio-posteriorly) smooth (Fig. 4a); medially ventral rim of clypeus near lower level of eyes (Fig. 2a); [carinae of posterior areola of propodeum lamelliform and parallel-sided (Fig. 4a)]	***R. longicaudatus*** Belokobylskij, 1994, **stat. nov.**

## Supplementary Material

XML Treatment for Rhysipolis longicaudatus

## Figures and Tables

**Figure 2a.**
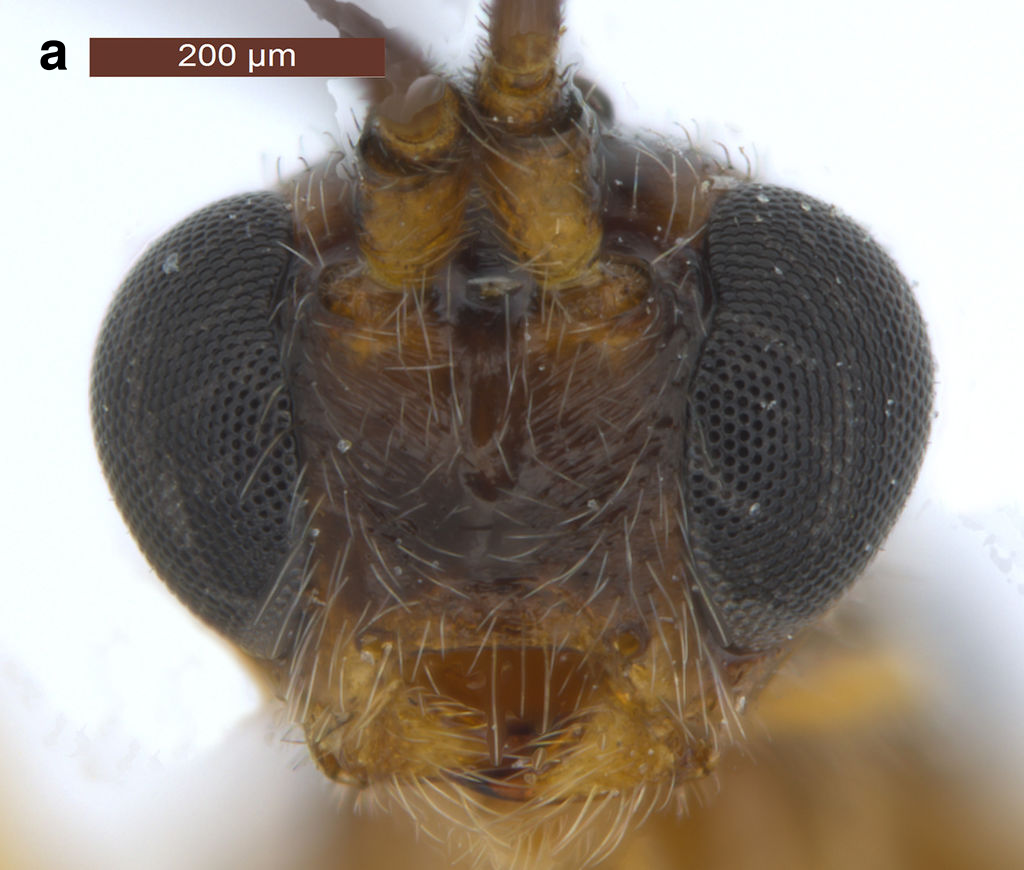
Head, anterior view

**Figure 2b.**
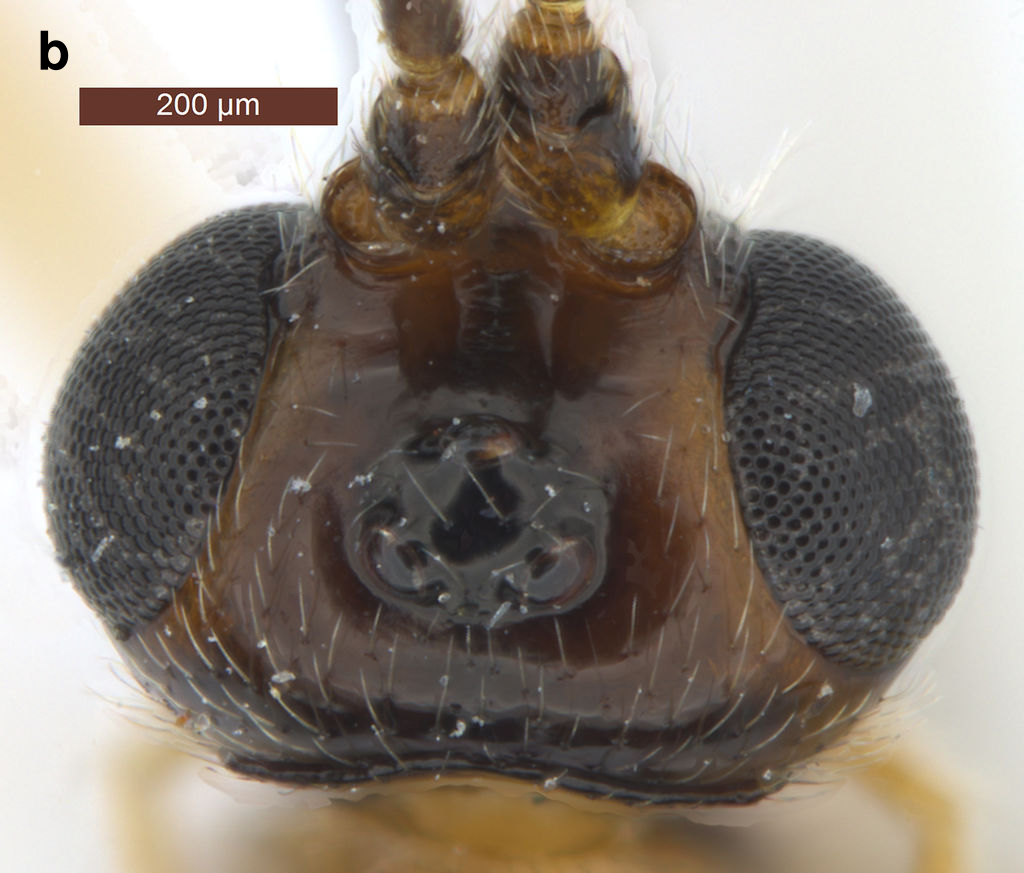
Head, dorsal view

**Figure 4a.**
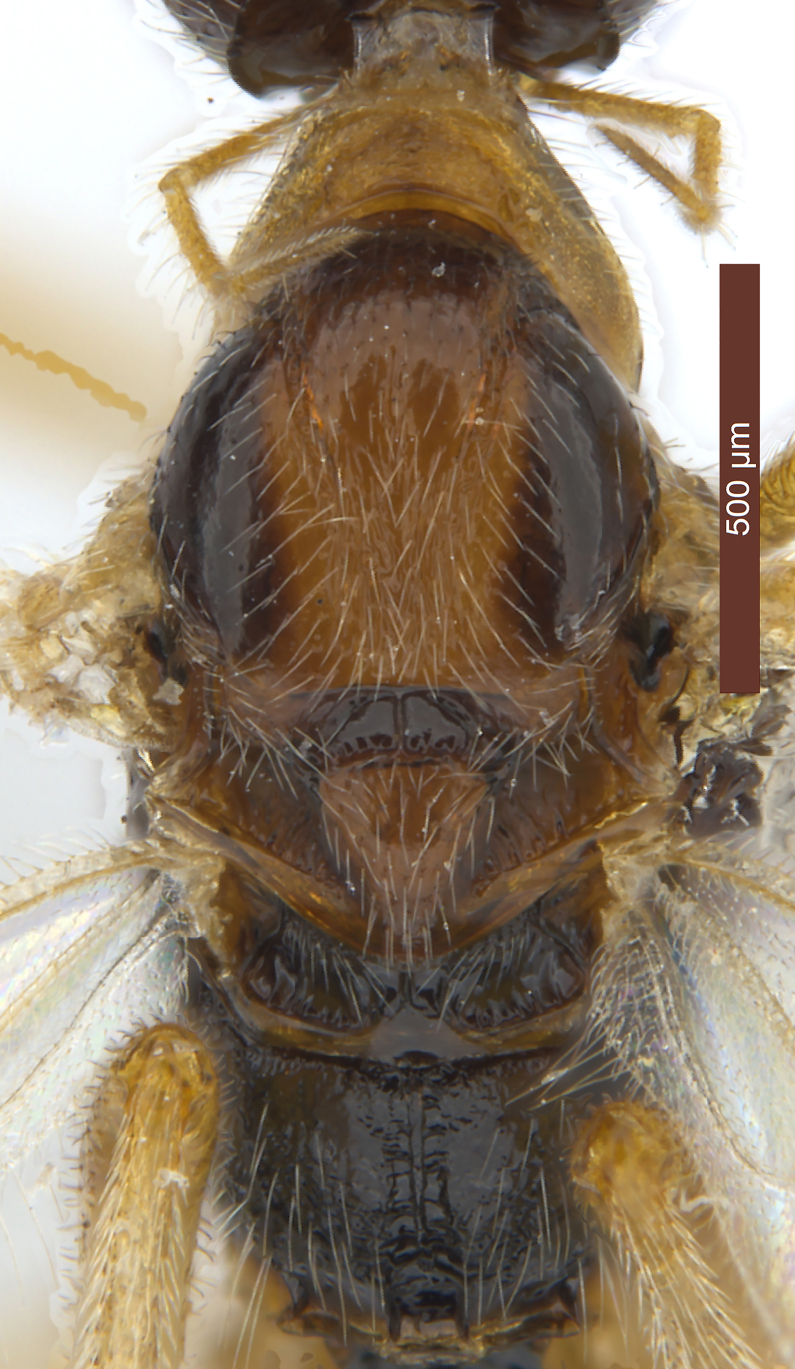
Mesosoma, dorsal view

**Figure 4b.**
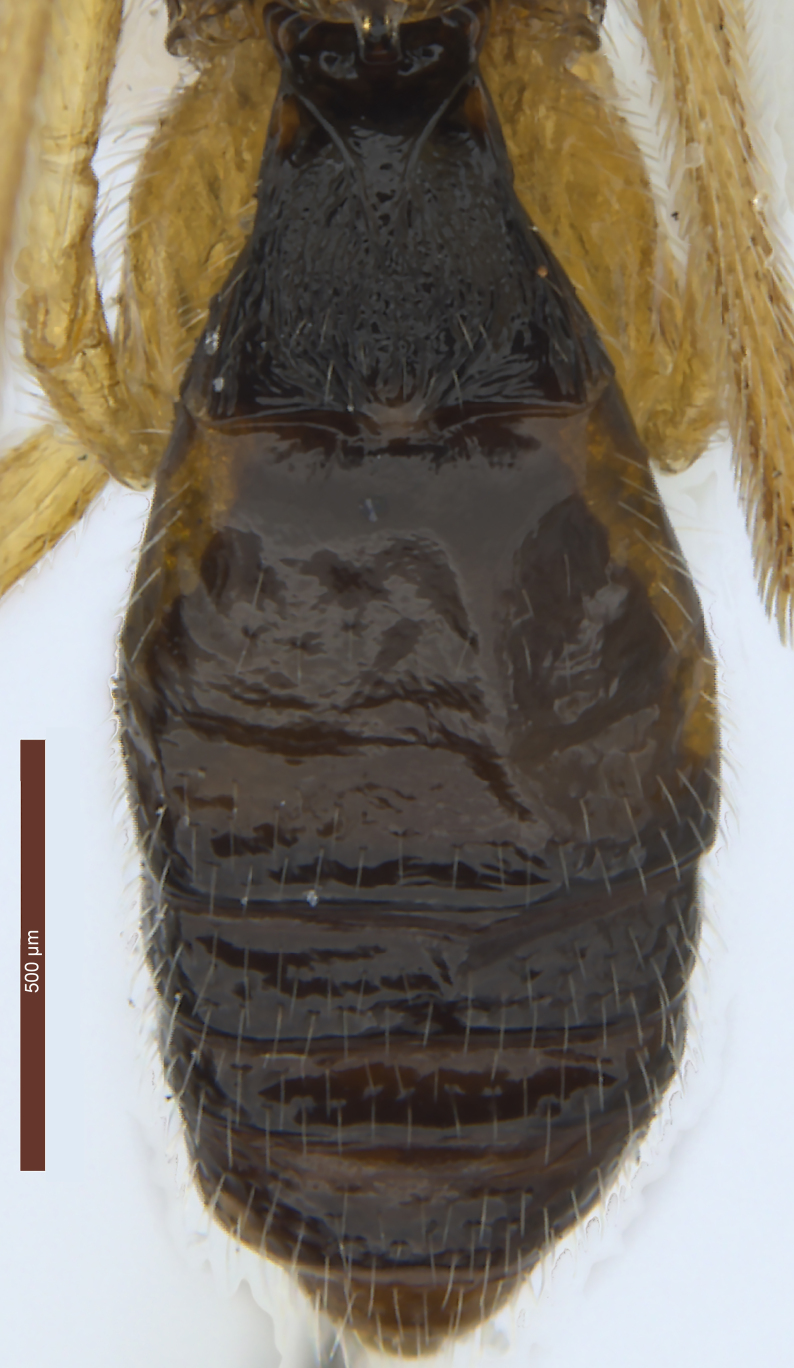
Metasoma, dorsal view

**Figure 5a.**
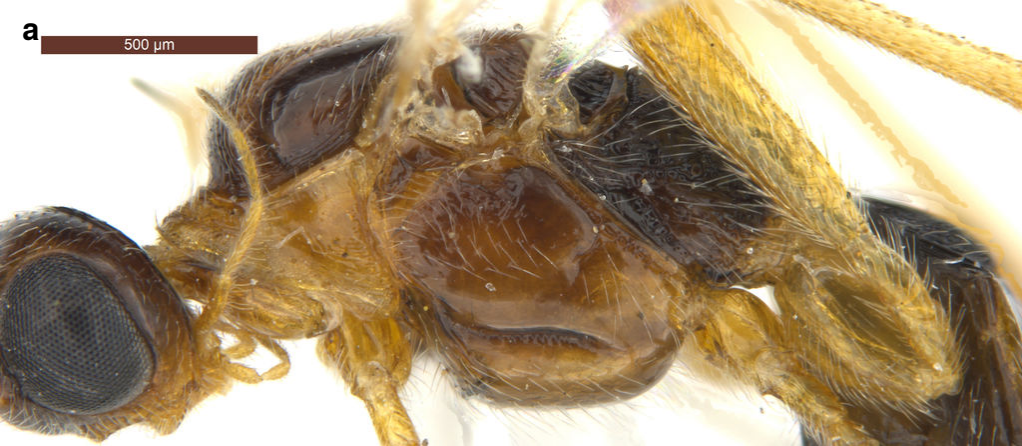
Mesosoma, lateral view

**Figure 5b.**
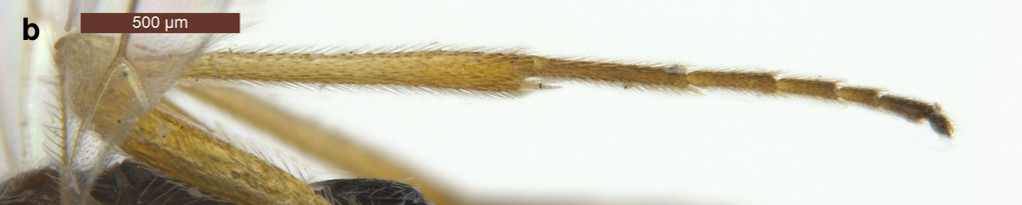
Hind tibia and tarsus, lateral view

**Figure 6a.**
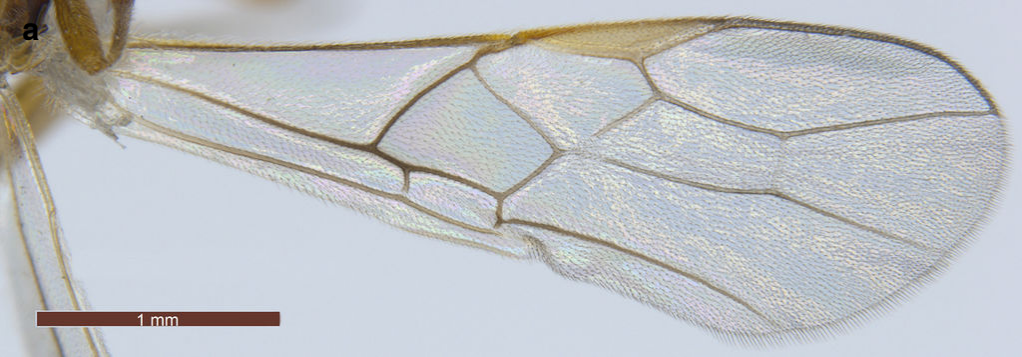
Fore wing

**Figure 6b.**
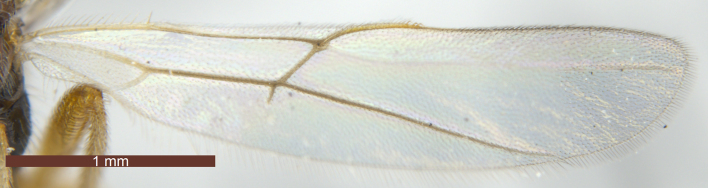
Hind wing

## References

[B2694473] Belokobylskij S A (1985). New species of Braconidae (Hymenoptera) from the Asiatic part of USSR and Mongolia. Entomologicheskoye Obozreniye.

[B2694483] Belokobylskij S A, Ler P A (1986). A new braconid species of the supertribe Exothecidii (Hymenoptera, Braconidae) from south of the USSR Far East. Systematika i Ekologija nasekomych Dalinego Vostoka. (Systematics and ecology of insects from the Far East).

[B2694507] Belokobylskij S A (1988). Braconids of the supertribe Exothecidii (Hymenoptera, Braconidae, Doryctinae) of Taiwan. Trudy Zoologicheskogo Instituta. Leningrad.

[B2694517] Belokobylskij S A, Kotenko A G (1994). A review of parasitic wasps of the subfamilies Doryctinae and Exothecinae (Hymenoptera, Braconidae) of the Far East (Eastern Siberia and neighbouring territories). Hymenopteran insects of Siberia and Far East: Memoirs of the Daursky Nature Reserve, no. 3.

[B2694541] Belokobylskij S A, Vu Q C (1988). Discovery of the genus *Rhysipolis* Förster (Hymenoptera, Braconidae) in the Indomalayan Region and description of a new species from Vietnam. Entomologicheskoye Obozreniye.

[B2694551] Gates M W, Heraty J M, Schauff M E, Wagner D L, Whitfield J B, Wahl D B (2002). Survey of the parasitic Hymenoptera on leafminers in California.. Journal of Hymenoptera Research.

[B2694583] Haliday A H (1836). Essay on parasitic Hymenoptera. Entomological Magazine.

[B2694603] Harris RA (1979). A glossary of surface sculpturing.. Occasional Papers in Entomology of the California Department of Food and Agriculture.

[B2694613] Marczak P, Buszko J (1994). Braconid wasps (Hymenoptera, Braconidae) reared from mining Lepidoptera.. Wiadomosci Entomologiczne.

[B2694623] Papp J (1987). Braconidae (Hymenoptera) from Korea. VIII. Acta Zoologica Hungarica.

[B2694633] Papp J (1996). Braconid wasps from the Cape Verde Islands (Hymenoptera, Braconidae) 1. Cheloninae, Exothecinae, Homolobinae, Microgastrinae, Rogadinae. Boletim do Museu Municipal do Funchal.

[B2694643] Scatolini D, Penteado-Dias A M, van Achterberg C (2002). *Pseudorhysipolis* gen. n. (Hymenoptera: Braconidae: Rhysipolinae) with nine new species from Brazil, Suriname and Panama.. Zoologische Mededelingen Leiden.

[B2694653] Shaw M R (1983). On evolution of endoparasitism: the biology of some genera of Rogadinae (Braconidae).. Contributions of the American Entomological Institute.

[B2694678] Shaw M R, Askew R R (1976). Ichneumonoidea (Hymenoptera) parasitic upon leaf-mining insects of the orders Lepidoptera, Hymenoptera and Coleoptera. Ecological Entomology.

[B2694688] Spencer L, Whitfield J B (1999). Revision of the nearctic species of *Rhysipolis* Förster (Hymenoptera: Braconidae). Transactions of the American Entomological Society.

[B2694698] Tobias V I (1976). Braconids of the Caucasus (Hymenoptera, Braconidae)..

[B2694707] van Achterberg C (1993). Illustrated key to the subfamilies of the Braconidae (Hymenoptera: Ichneumonoidea).. Zoologische Verhandelingen (Leiden).

[B2694717] Yu D S, van Achterberg C, Horstmann K Taxapad 2012–World Ichneumonoidae 2011. Taxonomy, Biology, Morphology and Distribution. On USB flash drive. Ottawa, Ontario, Canada. http://www.taxapad.com.

